# Reduction of Platelet Outdating and Shortage by Forecasting Demand With Statistical Learning and Deep Neural Networks: Modeling Study

**DOI:** 10.2196/29978

**Published:** 2022-02-01

**Authors:** Maximilian Schilling, Lennart Rickmann, Gabriele Hutschenreuter, Cord Spreckelsen

**Affiliations:** 1 Institute for Medical Informatics University Hospital Aachen RWTH Aachen University Aachen Germany; 2 Institute for Transfusion Medicine University Hospital Aachen RWTH Aachen University Aachen Germany; 3 Institute of Medical Statistics, Computer and Data Sciences Jena University Hospital Friedrich Schiller University Jena Germany

**Keywords:** platelets, demand forecasting, time series forecasting, blood inventory management, statistical learning, deep learning, LASSO, LSTM

## Abstract

**Background:**

Platelets are a valuable and perishable blood product. Managing platelet inventory is a demanding task because of short shelf lives and high variation in daily platelet use patterns. Predicting platelet demand is a promising step toward avoiding obsolescence and shortages and ensuring optimal care.

**Objective:**

The aim of this study is to forecast platelet demand for a given hospital using both a statistical model and a deep neural network. In addition, we aim to calculate the possible reduction in waste and shortage of platelets using said predictions in a retrospective simulation of the platelet inventory.

**Methods:**

Predictions of daily platelet demand were made by a least absolute shrinkage and selection operator (LASSO) model and a recurrent neural network (RNN) with long short-term memory (LSTM). Both models used the same set of 81 clinical features. Predictions were passed to a simulation of the blood inventory to calculate the possible reduction in waste and shortage as compared with historical data.

**Results:**

From January 1, 2008, to December 31, 2018, the waste and shortage rates for platelets were 10.1% and 6.5%, respectively. In simulations of platelet inventory, waste could be lowered to 4.9% with the LASSO and 5% with the RNN, whereas shortages were 2.1% and 1.7% with the LASSO and RNN, respectively. Daily predictions of platelet demand for the next 2 days had mean absolute percent errors of 25.5% (95% CI 24.6%-26.6%) with the LASSO and 26.3% (95% CI 25.3%-27.4%) with the LSTM (*P*=.01). Predictions for the next 4 days had mean absolute percent errors of 18.1% (95% CI 17.6%-18.6%) with the LASSO and 19.2% (95% CI 18.6%-19.8%) with the LSTM (*P*<.001).

**Conclusions:**

Both models allow for predictions of platelet demand with similar and sufficient accuracy to significantly reduce waste and shortage in a retrospective simulation study. The possible improvements in platelet inventory management are roughly equivalent to US $250,000 per year.

## Introduction

### Background

For blood centers, it is key to keep a balance between shortage and outdating of blood products to secure both cost efficiency and sufficient care for patients. This is especially true for short-lived blood products such as platelets. Forecasting demand has recently gained fresh attention as a way to address the problem, and the rise of *big data* and *artificial intelligence* in recent decades suggests new opportunities in this task [1,2].

Platelet transfusion is an indispensable part of modern medicine [3]. It is used prophylactically to reduce the risk of bleeding or therapeutically to manage active bleeding [3]. Most platelets are transfused to hematology and oncology patients, followed by patients undergoing severe surgical treatment [3-5]. In recent decades, a rise in platelet demand has been reported repeatedly [3,6-8].

As with other blood products, platelets need to be readily available at all times as demand might occur on short notice without obvious foreboding and timely transfusion is often critical [5]. Therefore, most blood centers try to store ample amounts of platelets and other blood products. However, the supply is limited by the number of donations.

Keeping sufficient stock is especially difficult with platelets because of their short shelf life of 5-7 days, including time for preparation and quality control [9]. A large stock may lead to large amounts of wastage because of outdating, whereas a slender stock increases the risk of shortages [10,11]. Platelet outdating rates are the highest of all blood products and are typically reported at 10% to 20% [6,11].

In a recent systematic review, Flint et al [11] provided a detailed overview of existing methods to reduce platelet outdating, one of which was forecasting platelet demand. By forecasting demand, production can be adjusted accordingly to reduce both outdating and shortage. It has been stated that prediction and modeling will have increasingly important roles in managing blood inventory [12]. However, to this day, there are very few scientifically published approaches to forecasting platelet demand [11].

Several authors have investigated different univariate time series models to predict platelet demand, including moving averages, weighted moving averages, exponential smoothing, Winters models, and autoregressive moving averages (ARIMA) [10,13-15]. Fanoodi et al [14] reported improved prediction when using univariate time series modeling by means of an artificial neural network (ANN) compared with an ARIMA model.

More recent studies have included additional clinical data as predictors in multivariate models [1,2,16]. Khaldi et al [16] predicted the monthly demand of platelets, red blood cells, and plasma by means of a multivariate ANN with a total of 10 features, including census data, number of traffic accidents per day, and clinical events such as hemorrhage and deliveries at risk. They reported better prediction accuracy for the ANN compared with a univariate ARIMA model.

Guan et al [1] presented the first *big data* approach to predict platelet demand for the next 3 days and minimize wastage at the Stanford Blood Centre. The authors used 43 features, including hospital census data, complete blood count, day-of-the-week status, and average daily transfusions over the previous 7 days to predict platelet demand [1]. They included the predictions in a linear optimization problem similar to the least absolute shrinkage and selection operator (LASSO) method that also accounted for the structure of the platelet inventory and testing procedure at Stanford Blood Centre to directly minimize wastage [1]. Comparing their findings with retrospective data over 29 consecutive months, Guan et al [1] found that the introduction of such a model in their institution could lower outdating from 10.3% to 3.2% with no shortages.

During the course of this study, Motamedi et al [2] published a study comparing multiple univariate and multivariate models to predict daily platelet demand at Canadian Blood Services: ARIMA, Prophet, LASSO, and a long short-term memory (LSTM) network. They compared the models in terms of prediction errors measured by root mean squared error (RMSE) and mean absolute percent error (MAPE) with 2 and 8 years of training data. The multivariate models (LASSO and LSTM) consistently outperformed univariate time series (ARIMA and Prophet), especially on the shorter training sets. The LASSO performed best, with the LSTM being marginally worse. For the multivariate models, the authors included hospital census data, complete blood count, day-of-the-week status, average transfusions over the previous 7 days, and number of transfusions on the previous day as possible predictors. The features for both the LASSO and the LSTM were selected by the LASSO.

According to the current state of the art, LASSO and LSTM networks seem to be very promising models for the prediction of platelet demand. However, the accuracy of any prediction model may vary between different sites because of the amount and quality of the available data. Furthermore, it is unclear how accurate a prediction needs to be to enable an actual reduction in waste and shortage. This may also vary between sites supposedly because of differences in their respective blood inventories, such as shelf life of platelets, average daily transfusion volume, production and quality control practices, or availability of donations.

### Objective

Therefore, the aims of this study are 2-fold: the first aim is to predict daily platelet demand at the RWTH Aachen University Hospital (UKA) using both a LASSO and an LSTM network. The second aim is to design a simulation model of the blood inventory at UKA, establish an ordering strategy based on the predictions, and quantify possible reductions in waste and shortage rates as compared with retrospective data. To the best of our knowledge, this is the first study to compare these 2 models in terms of both prediction accuracy and possible reduction in waste and shortage rates based on prediction-driven simulations.

## Methods

### General Approach

**Figure 1:**
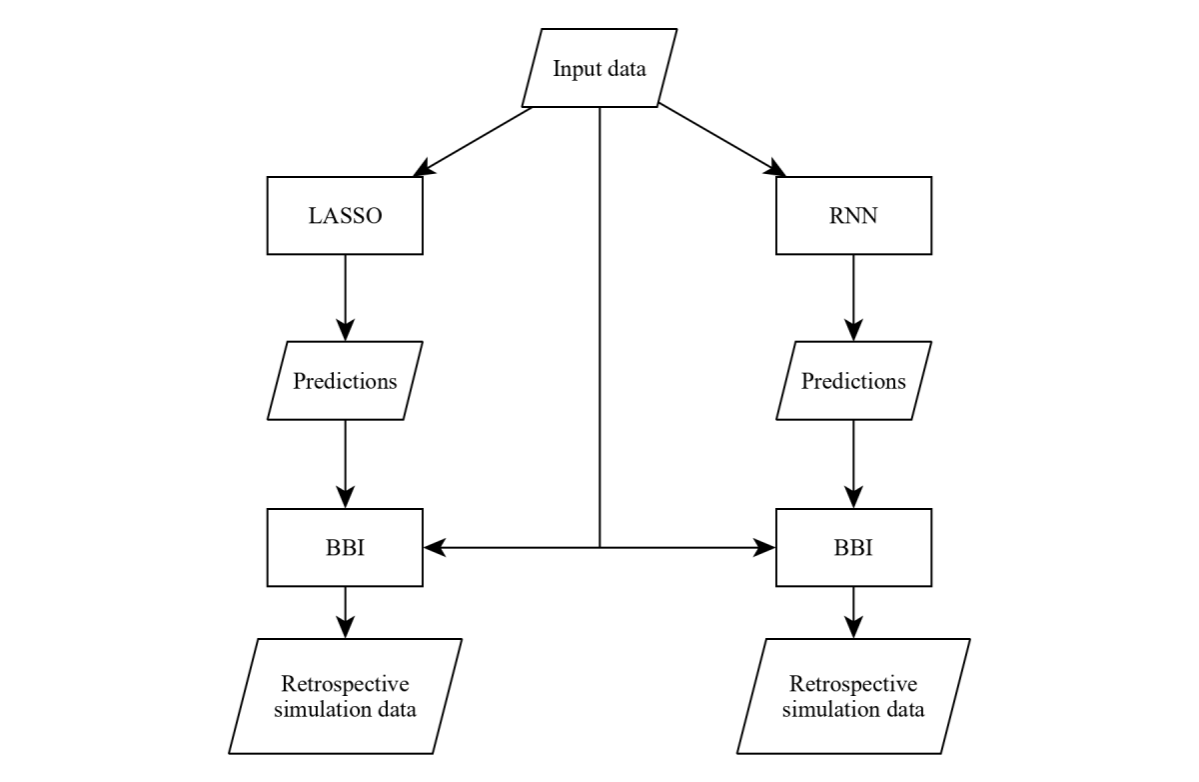
General approach: input data are fed to 2 separate prediction models—least absolute shrinkage and selection operator and recurrent neural network. Predictions of platelet demand are passed to a simulation model of the blood bank inventory. Possible reductions in waste and shortage rates are calculated in comparison with retrospective data. BBI: blood bank inventory; LASSO: least absolute shrinkage and selection operator; RNN: recurrent neural network.

According to the aims of this study, our approach was 2-fold (Figure 1). First, we used retrospective data from the UKA electronic health record (EHR) to build 2 separate prediction models for platelet demand: a LASSO model and a deep learning recurrent neural network (RNN) with an LSTM layer. Second, we designed a simulation model of our blood bank inventory. Combining both parts, forecasts of platelet demand were passed to the blood bank inventory to prematurely adjust platelet production and calculate the resulting expiration and shortage rates in a retrospective simulation study.

### Data Acquisition

All data were sourced from the UKA EHR. No personal patient data were used. The local ethics committee approved the data acquisition and analysis (code EK282/19). For the period from January 1, 2008, to December 31, 2018, we obtained data in three categories: (1) platelet ingoings and outgoings as recorded by the transfusion department; (2) census data for all wards, outpatient clinics, and operation rooms; and (3) complete blood count.

### Data Cleaning and Preparation

Data were obtained as a daily time series and aggregated in a single database. Platelet ingoings and outgoings were grouped by source (in-house production and purchase) and disposition (use, waste, sales, and quality control) and documented as platelet units per day. Census data were documented as patients per day grouped by inpatient clinics, outpatient clinics, surgeries, and planned surgeries for the next day and subgrouped by department. Complete blood count data other than platelet count were documented as the number of measurements out of the norm per day. Platelet count was recorded as the number of measurements per day within specific intervals with regard to platelet transfusion guidelines: <5/nL, 5-10/nL, 10-20/nL, 20-50/nL, 50-70/nL, 70-100/nL, and 100-150/nL [17-20].

Within the UKA EHR, zeroes (eg, no platelets transfused on a given day) are not documented and are represented as missing values. Therefore, we used zeroes to represent the missing values rather than applying imputation. The only exception is census data, where a missing value might indicate that the given department did not exist at that point. Therefore, all departments that did not continually exist throughout the examined 10-year period were excluded. All census data with <400 nonzero values were excluded as it was assumed that these time series did not contain significant information. During the initial inspection of the data, we found that a considerable amount of platelet traffic data was mislabeled in terms of disposition. Over the years, changing collaborations with other clinics and local practices as well as a change in the inventory software have resulted in inconsistent data labeling. A particular problem here was the units that were given to partner clinics but labeled as used in-house rather than sold. Therefore, all platelet traffic data were systematically verified for correct labeling. Mislabeled data were corrected if possible and excluded otherwise. Less than 1% (305/46,205, 0.66%) of the total transfusion records were excluded because of this problem. The entire data set is provided in Multimedia Appendix 1.

### Included Predictors

All features from the census and complete blood count data with a correlation of *r*^2^≥0.2 to platelet use were included as predictors in the prediction models. Previous studies have shown that platelet transfusion shows a strong pattern of autocorrelation and is dependent on the day of the week [1,10,13]. Therefore, the average number of transfusions per day over the previous 7 days and the day-of-the-week dummy variables were added to the models. Thus, a total of 81 possible features were included for prediction.

### Blood Bank Inventory Model

The UKA transfusion department collects and prepares platelets by apheresis. Registered donors have regular appointments or are called in individually for donation. The entire production chain, including donor activation, platelet preparation, and quality control, takes 2 days (1 day for donor activation and 1 day for preparation and quality control). Donors are only called on Monday through Friday. Therefore, no fresh platelets arrive on Sundays or Mondays. After quality control, platelets have a remaining shelf life of 4 days. In case of slender stock, additional platelets are purchased from other hospitals or local providers such as the local section of the German Red Cross Society. Such an *emergency purchase* is available approximately 2 hours after order. In rare cases, UKA sells platelets to other clinics with a short supply if stock is high. However, as sales occur both very rarely and irregularly, they were not included in the model.

For retrospective simulations of the blood bank inventory, production orders, purchases, discards, and stock are calculated at the end of each day of the observation period using an iterative approach. The stepwise calculation model described below was recalculated for each day of the time series.

As no fresh platelets arrive on Sundays and Mondays, different ordering strategies and prediction intervals for demand are required for different days of the week. Platelets ordered on day *i* between Sunday and Wednesday will arrive on day *i* + 2. Therefore, these orders need to countervail all platelet outgoings on day *i* + 1 and *i* + 2. Orders made on Thursdays also arrive after 2 days but need to account for the demand of the next 4 days as no orders can be made on Fridays and Saturdays. Considering current stock as well as preceding orders, we established the ordering strategy given in Equation 1, where *o_i_* is the number of platelets ordered on day *i*, *α* is the parameter target value for platelet stock at end of day, *s_i_* is the current platelet stock at the end of day *i*, *p_i_*(2) is the predicted demand for days *i* + 1 and *i* + 2, *p_i_*(4) is the predicted demand for the next 4 days, and *o_i–1_* is the number of units ordered on day *i* – 1 as these will arrive on day *i* + 1. *d_w_*(*i*) represents the weekday status of day *i*, with values starting from 0 for Sundays to 6 representing Saturdays.



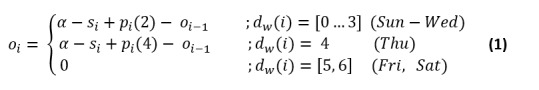



**Figure 2:**
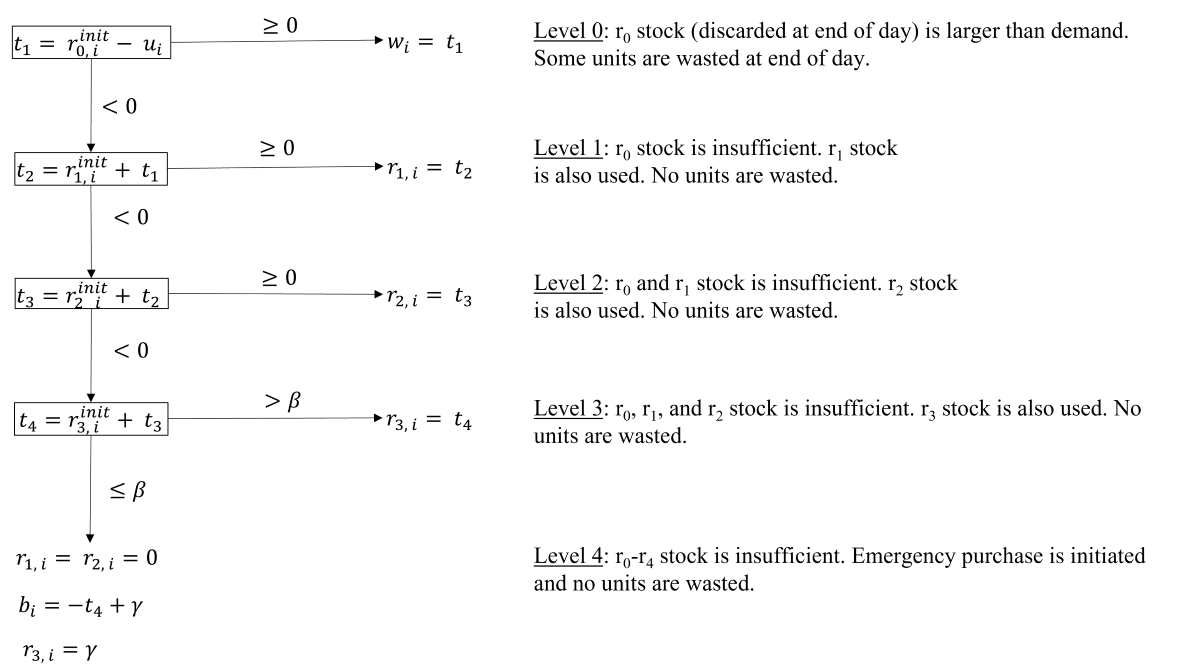
Blood bank inventory stepwise calculation model. For each day of the time series, initial values are set according to Equations 2-7. This stepwise calculation is then carried out and, finally, total stock at end of day is calculated according to Equation 8.

We established the stepwise calculation model shown in Figure 2 to calculate *s_i_* as well as other inventory variables. Here, *r_x, i_* represents the remaining units that will be discarded at the end of day *i* + *x*, *x* being the remaining shelf life, with values ranging from 0 to 3 (0 indicating that these units are discarded at the end of that same day). *u_i_* is the number of platelets actually used on day *i*, *w_i_* is the number of platelets wasted on day *i*, and *b_i_* is the number of units purchased from other providers on day *i*. *β* and *γ* are parameters to control for emergency purchases—a purchase is made if stock falls to or below *β* and, in this case, *γ* is the target value for stock after emergency purchase. *t1*, *t2*, *t3*, and *t4* are temporary variables for convenient display. We assume that the oldest platelet units are always the first to be used. The following defaults (indicated as such by the notion *init*) are set each day before moving through the calculation:



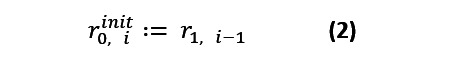





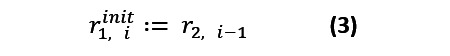





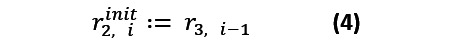





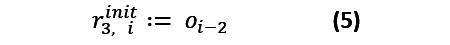





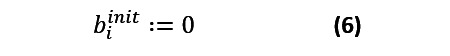





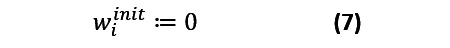



After moving through the stepwise calculation, *s_i_* is calculated to


*s_i_ = r_1, i_ + r_2, i_ + r_a, I_*
**(8)**

*α*, *β*, and *γ* are chosen by minimizing the total cost *c* as defined by Equation 9 using an exhaustive grid search with a range from 0 to 30 and steps of 1:



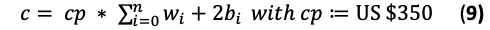



We arrived at this definition because the cost for a single platelet unit is approximately US $350 when produced locally and planned in advance. Buying platelets in an emergency is more expensive. The actual price varies widely depending on several factors, such as the total amount bought and costs for transportation. On average, the price of a platelet unit bought in an emergency is almost double compared with preplanned production. The weight in Equation 9 was rounded up to also punish the possible delay in transfusion because of transportation time. Note that the blood bank inventory allows for temporarily negative values for stock when moving through the stepwise calculation process given in Figure 2 (*t1*, *t2*, *t3*, and *t4*). Therefore, values of 0 for *β* and *γ* are possible. In this case, emergency purchase is only initiated when demand exceeds stock (*β*=0), and just enough units are bought to satisfy demand, ending that day with empty stock (*γ*=0). It is assumed that emergency purchases will always succeed and, therefore, it is simply a matter of buying as many units as required in circumstances where there is no platelet stock. Consider the following example for *β*=*γ*=0: stock is 2, and there is an unexpected need for 4 platelet units (*t4*=−2). Emergency purchase is initiated because *t4*<*β*, and 2 units are bought because *b_i_* = −*t4* + *γ* = 2 + 0. The 2 units from stock and the 2 units from emergency purchase are transfused, and the stock after purchase is 0 (*γ*=0).

### Prediction Model Setup and Validation Strategy

Standard supervised learning was used to predict platelet demand for the next 2 and 4 days. Predictions were made using rolling-origin-recalibration evaluation as described by Bergmeir and Benítez [21]. First, the models were trained on the first 500 days of the time series. Predictions were made for days 501 to 528. The models were then retrained on the first 528 days, and the next predictions were made for the following 28 days. Both models were retrained in this fashion every 28 days, including recalibration of all hyperparameters. To this end, we also followed the recommendations of Bergmeir and Benítez [21] using 5-fold blocked cross-validation and the augmented Dickey–Fuller unit root test with a trend-corrected regression to check for stationarity in the presence of a trend over time. The interval of 28 days was chosen to account for the weekly seasonality in the data while controlling for the computational expense of repeated retraining [1,10,13]. Mean squared error (MSE) was used as a loss function for the cross-validation. We used the Python 3 language library scikit-learn (Python Software Foundation) to implement this validation strategy [22].

The accuracy of the predictions was measured with RMSE, the Pearson correlation coefficient of the predicted and true values (*r*^2^), and MAPE and expressed as mean and 95% CIs. CIs were calculated using bootstrapping [23]. *P* values for the differences in RMSE and MAPE between the models were obtained from the corresponding CI as described by Altman et al [24]. *P*<.05 was defined as statistically significant.

### Statistical Model

The first model was a LASSO as described by Tibshirani [25]. The LASSO is a shrinkage model for multiple linear regression. Regression coefficients are calculated by minimizing the residual sum of squares with a sparsity penalty given by the L1 norm of the coefficient vector multiplied by a tuning parameter. Owing to the form of the constraint, all coefficients are shrunken toward 0, and some become exactly 0. In this way, the LASSO trades off variance for bias while also performing variable selection and producing interpretable models [25]. As described above, the tuning parameter was chosen via 5-fold blocked cross-validation with MSE as the loss function. We used the Python 3 language library scikit-learn to implement this model [22].

### Deep Learning Model

**Figure 3:**
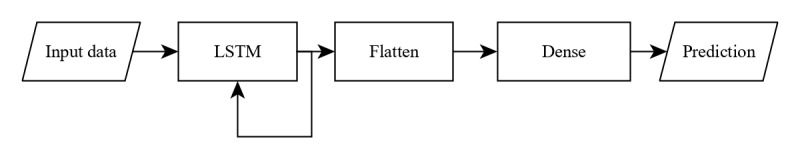
Architecture of the recurrent neural network used for prediction of platelet demand. Data are first passed to a long short-term memory layer followed by a flatten layer and a dense layer to generate an integer output to our regression problem. LSTM: long short-term memory.

The second prediction model was an RNN. We used a sequential model from the TensorFlow (Google Brain Team) library (Figure 3) [26]. The first layer was an LSTM as described by Hochreiter and Schmidhuber [27]. An L1–L2 regularizer was combined with a dropout rate to reduce overfitting. The LSTM output was passed to a flatten layer. We treated the prediction of platelet demand as a regression problem and, therefore, used a dense layer with a linear activation function. The dense layer consisted of a single neuron. In preliminary tests on the data, the dropout rate, L1–L2 regularization, batch size, activation function in the flatten layer, and number of units in the LSTM layer were identified as influential hyperparameters. Therefore, they were adjusted during training using a randomized grid search within the validation strategy described above. All hyperparameters and their search spaces are summarized in Table 1. We used TensorFlow and the Python 3 language library Keras to implement this model [26,28].

**Table 1:** Hyperparameters of the deep learning model and their respective search space for optimization via randomized grid search.

Parameter	Search space
Batch size	50, 100
LSTM^a^ units	10, 50
Dropout rate	0%-50%, steps of 5
L1 regularizer	10^−9^, 10^−7^, 10^−5^, 10^−3^
L2 regularizer	10^−9^, 10^−7^, 10^−5^, 10^−3^
Flatten layer activation function	ReLU^b^, linear

^a^LSTM: long short-term memory.

^b^ReLU: rectified linear unit.

## Results

### Platelet Transfusion, Outdating, and Shortage

**Figure 4:**
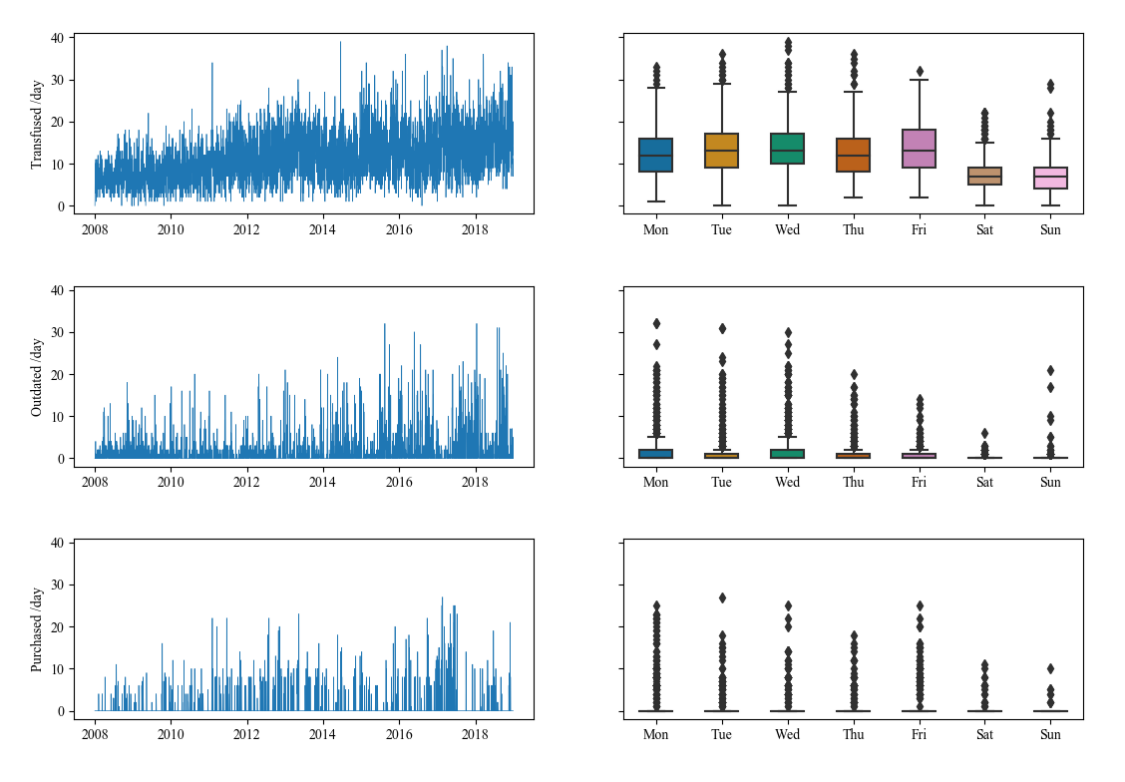
Top to bottom: transfusions, outdating, and emergency purchase of platelet units. Left: daily patterns. Right: averages by day of the week.

During the observed period, 46,205 platelet units where transfused at UKA. Daily transfusions ranged between 0 and 39 with an average of 11.50 (SD 6.02). Units transfused per year increased from 2566 in 2008 to 5891 in 2018. Daily averages were significantly different for different days of the week as determined by 1-way analysis of variance (ANOVA; *F*_6_=187; *P*<.001; Figure 4). No significant difference was found for month of the year, also by 1-way ANOVA (*F*_11_=1.56; *P*=.10). More platelets were transfused during the week than on weekends. The time series of daily platelet transfusions was confirmed to be trend-stationary by augmented Dickey–Fuller unit root test with a trend-corrected regression (augmented Dickey–Fuller statistic=−8.34; *P*<.001).

A total of 4654 platelet units expired during the observed 10 years. The daily average expiration was 1.16 (SD 2.77, range 0-32). Furthermore, 1-way ANOVA showed significant differences in daily platelet expiration across different days of the week (*F*_6_=48.9; *P*<.001), with higher values during the week than on weekends (Figure 4). There was no significant difference across the months of the year (*F*_11_=1.34; *P*=.20). The expiration rates relative to transfusions were 10.1% and 11% for the entire observed period and the validation period, respectively.

Emergency purchases were made for a total of 2988 units, with a daily mean of 0.74 (SD 2.77, range 0-27). Furthermore, 1-way ANOVA showed significant differences in daily platelet purchases across different days of the week (*F*_6_=28.6; *P*<.001; Figure 4) as well as across the months of the year (*F*_11_=1.82; *P*=.046). Platelet supply was more often short during the week than during weekends, with most emergency purchases being on Mondays. February and June were the months with the most severe supply shortages. The shortage rates relative to transfusions were 6.47% and 7.05% for the entire observed period and the validation period, respectively.

### Blood Bank Inventory Simulation

**Figure 5:**
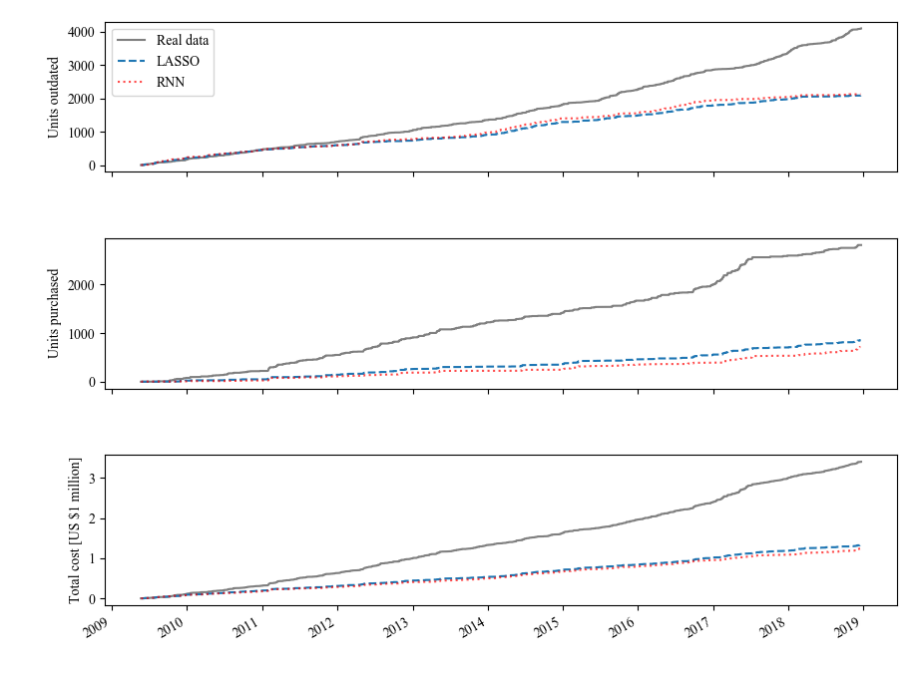
Simulated cumulative outdating, purchase, and cost (as defined by Equation 9) compared with retrospective data. LASSO: least absolute shrinkage and selection operator; RNN: recurrent neural network.

The retrospective simulations of our blood bank inventory using the above-described blood bank inventory and prediction models yielded the results described in this section. Blood bank inventory simulation was performed separately for predictions made by the LASSO and RNN models. Simulated outdating rates were similar for both prediction methods, whereas purchase and overall cost as defined by Equation 9 were lower with the RNN forecasts. With the LASSO, outdating and shortage were reduced from 11% to 4.93% and from 7.05% to 2.11%, respectively. Using the predictions of the RNN, outdating was reduced to 5%, and shortage fell to 1.68%. These reductions in outdating and shortage are roughly equivalent to savings of US $250,000 per annum. Simulated total cost was US $1.33 million with the LASSO and US $1.241 million with the RNN (Equation 9). Figure 5 shows the cumulative plots for outdating, purchase, and overall cost for both prediction models compared with the real retrospective data.

The target values for platelet stock at the end of each day (*α*) were calculated to be 13 and 14 when using the LASSO and RNN predictions, respectively. The threshold for emergency purchase of platelets (*β*) as well as the target value for platelet stock after such purchases (*γ*) were 0 for both models. Note that the blood bank inventory allows for temporarily negative values for stock when moving through the stepwise calculation given in Figure 2 (*t1*, *t2*, *t3*, and *t4*). Therefore, values of 0 for *β* and *γ* mean that emergency purchases are only initiated when demand exceeds current stock (*β*=0) and that just enough units are bought to satisfy demand, ending that day with empty stock (*γ*=0).

### Forecast Accuracy

Table 2 shows the forecast accuracy for predictions of platelet demand for the next 2 and 4 days measured by RMSE (the square root of the mean square deviation of the predicted values from the true values), the Pearson correlation coefficient of the predicted and true values (*r*^2^), and MAPE for both the LASSO and RNN models. The LASSO performed slightly better than the RNN in terms of these error measures. The differences were statistically significant only for RMSE and MAPE for the 4-day forecast.

**Figure 6:**
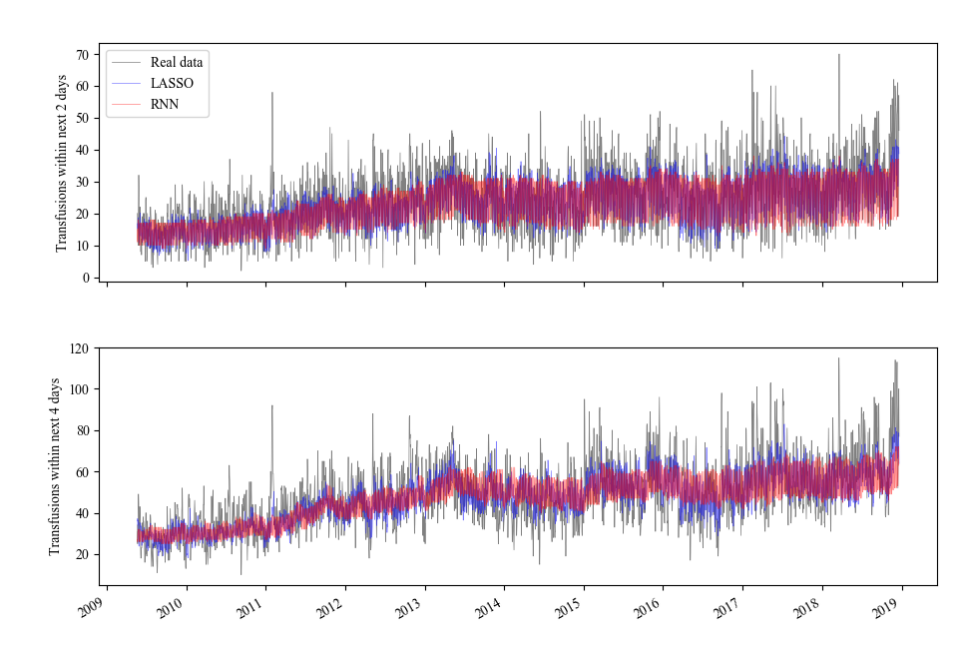
Longitudinal time series plots of demand predictions and real values of platelet demand. LASSO: least absolute shrinkage and selection operator; RNN: recurrent neural network.

Figure 6 shows longitudinal plots of predicted platelet demand alongside the true values for both models and both prediction tasks. Both models trade off variance for bias in their predictions—the RNN more so than the LASSO but with very similar results, as can be seen in Table 2.

**Table 2:** Forecast performance of the least absolute shrinkage and selection operator (LASSO) and recurrent neural network (RNN) for predictions of platelet demand for the next 2 and 4 days.

Forecast period and method			RMSE^a^ (95% CI)		*P* value			*r*^2b^ (95% CI)			*P* value			MAPE^c^ (%; 95% CI)			*P* value	
**Next 2 days**						.09						.88						.10
	LASSO	6.77 (6.57-6.98)					0.73 (0.71-0.74)						25.51 (24.56-26.51)					
	RNN	6.94 (6.74-7.15)					0.71 (0.70-0.73)						26.32 (25.33-27.41)					
**Next 4 days**						<.001						.07						.001
	LASSO	10.78 (10.46-11.13)					0.74 (0.72-0.75)						18.11 (17.59-18.61)					
	RNN	11.52 (11.17-11.87)					0.69 (0.67-0.71)						19.22 (18.46-19.82)					

^a^RMSE: root mean squared error.

^b^Pearson correlation coefficient of the predictions and the true values.

^c^MAPE: mean absolute percent error.

### Predictors of Platelet Demand

As described above, the LASSO performs feature selection and produces interpretable models. The most influential predictors of platelet demand for the next 2 and 4 days are listed in Table 3. The strongest predictor in both prediction tasks was the average number of platelet transfusions over the previous 7 days. Other influential predictors were day of the week, number of platelet counts between 20/nL and 10/nL, patients in the oncology and psychiatry departments, and surgeries planned for the next day in the neurosurgery department. The average number of nonzero predictors over all model iterations was 50.7 (SD 20.409) and 41.8 (SD 14.389) in the 2-day and 4-day forecasts, respectively. Owing to its complex layered structure, the RNN does not provide direct information on the influence of individual predictors.

**Table 3:** Strongest predictors of platelet demand in the least absolute shrinkage and selection operator model. Mean predictor weights over all model iterations.

Forecast and predictor		Predictor weight, mean (SD)
**2-day forecast**		
	PL7^a^	3.04
	Weekday Friday	−2.12
	Weekday Thursday	−2.08
	I4^b^	1.54
	Weekday Saturday	−1.17
	CBC_PL_cont 20-10^c^	1.17
	PP^d^	0.99
	OP_P_NC^e^	0.99
**4-day forecast**		
	PL7	1.68
	Weekday Saturday	−1.14
	Weekday Friday	−1.01
	CBC_PL_cont 20-10	0.80
	I4	0.64
	OP_P_NC	0.61
	PP	0.60
	OP_P_GG^f^	0.60

^a^PL7: platelet transfusions over previous 7 days.

^b^I4: number of patients in the oncology ward.

^c^CBC_PL_cont 10-20: daily number of complete blood count essays with platelet count between >10/nL and ≤20/nL.

^d^PP: number of patients in the psychiatry wards.

^e^OP_P_NC: number of planned surgeries for the next day in the neurosurgery department.

^f^OP_P_GG: number of planned surgeries for the next day in the vascular surgery department.

## Discussion

### Principal Findings

The results of this study show that it is possible to predict platelet demand at UKA with high accuracy using both approaches investigated: LASSO and RNN with LSTM. These results confirm previous work and, as a particularly relevant aspect, support the generalizability of these models to different sites [1,2].

Furthermore, the simulations of the blood bank inventory suggest that these predictions can be used to reduce waste and shortage of platelets at UKA by a considerable amount. The implementation of such a prediction system at UKA might lead to savings as high as US $250,000 per year. Although several studies have investigated the prediction of platelet demand, very few have examined the extent to which these predictions can be used to improve inventory management via simulations or field tests [1,2,10,13-16]. To the best of our knowledge, this study is the first to compare LASSO and LSTM models in terms of both prediction accuracy and possible reduction in waste and shortage rates based on prediction-driven simulations.

Both the LASSO and RNNs with LSTM have previously been described as powerful tools for predicting platelet demand [1,2]. Motamedi et al [2] predicted the next-day platelet demand using these models, with very similar results to our study. They reported MAPE values of 28.02% and 28.52% for the LASSO and LSTM, respectively. Guan et al [1] reported possible reduction in platelet outdating from 10.3% to 3.2% with no shortages when using predictions made with the LASSO. However, they did not report the prediction accuracy of their model.

The prediction accuracy of the RNN was marginally inferior to that of the LASSO in our study. This was previously reported by Motamedi et al [2]. However, we argue that the use of deep learning holds great potential not yet fully explored by our project. The most important point is the ability of deep neural networks to take in much more heterogeneous data than a statistical model such as the LASSO [29]. Inclusion of data such as diagnosis and medical history of patients may lead to further refinement of predictions. Despite this potential, the fact that neural networks do not allow for simple interpretation of influential predictors, often referred to as the *Black Box Problem*, is a potential downside of these systems [29-31].

The most influential predictors identified by the LASSO (Table 3) were largely in accordance with previous studies. Previous transfusions and day of the week, the most important predictors in our model, have been described as influential by several authors [1,2,10,13]. In addition, Guan et al [1,2], who also used the LASSO, reported great influence for red cell count and number of patients in the neurosurgery, vascular, and trauma departments. Motamedi et al [2] reported high influence of previous use, day of the week, and abnormal platelet count in their LASSO model. Interestingly, neither of these studies found the number of patients in the hematology and oncology departments to be an influential predictor despite the fact that platelet transfusions are very common in these patients [1-5]. However, this may be due to the intercorrelation effects of the predictors.

As somewhat of an unexpected finding, we observed that the blood bank simulation provided better results in terms of total cost and shortage rates when using RNN predictions, whereas, in accordance with previous results, the predictions made with the LASSO were slightly better in terms of RMSE, *r*^2^, and MAPE than those of the RNN. Although the differences are small, this indicates that these error measures might not be ideal for the problem. More specifically, the design of the ordering process, as formalized in Equation 1, allows for bias in the predictions to be compensated by the target value for the end-of-day stock (*α*). However, the variance in prediction errors cannot be compensated. Furthermore, because of the platelets’ shelf life of 4 days, prediction errors can be (randomly) compensated to some extent by opposing errors within 4 days. Finally, our definition of total cost (Equation 9) punishes shortage more severely than an excess of platelets. These aspects are not adequately represented by error measures such as RMSE, *r*^2^, or MAPE. In particular, the temporal sequence of errors was not accounted for.

Therefore, we might be missing out on some further reduction in waste and shortage rates by using MSE as a loss function to train the prediction models. Guan et al [1] circumvented this problem by translating demand predictions and modeling of the blood bank inventory into a single optimization problem, thereby using outdating of platelets as a loss function. The problem could also be addressed by replacing MSE as a loss function with error measures that are specifically adapted to the problem at hand. Moreover, this highlights the need for inventory simulation or field tests for any prediction model as the potential to reduce waste and shortage rates is to some extent dependent on the structure and processes of the blood inventory. Further investigation is needed in this area.

### Limitations and Next Steps

With the aforementioned in mind, the modular structure of our system with the prediction models and the blood bank inventory as independent components is a limitation of our study. However, it also has several advantages. First, it reduces the complexity of the overall system. On the one hand, this allows for simple interpretation and comparison of the prediction models. In contrast, it enables the modeling of a very complex blood inventory, incorporating separate predictions for weekdays and weekends as well as emergency purchases while keeping training times and computational expense manageable as the prediction models do not need to be retrained during the grid search for ideal blood bank inventory parameters. This flexible modular approach will also allow for the addition of further modules, such as a component accounting for blood types in the predictions.

The absence of such a module in our system is another limitation of this study. Although relevant to platelet transfusion, our forecasts do not account for ABO blood types and Rh status [18,32]. There is very limited literature on incorporating blood types in predictions of platelet demand. Critchfield et al [13] used a 7-day moving average of type distribution to account for ABO blood types. Fanoodi et al [14] treated each blood type (ABO and Rh status) as an independent time series for prediction. Although this method is straightforward, it reduces the number of data points available to the prediction models and might lead to reduced prediction accuracy. We suggest the addition of a separate prediction model to our system to forecast blood type distribution of demand. The strong pattern of autocorrelation in platelet demand, supposedly caused by the fact that most patients receive several transfusions over a prolonged period, suggests that the distribution of blood types might also show strong autocorrelation [10,13]. The distribution of blood types in the population could be a further clue to address this problem. Another option is to directly include blood types in a deep learning model based on the RNN presented here as these models are capable of performing complex end-to-end prediction tasks [29].

Although RMSE and MAPE are commonly used in the evaluation of time series forecasts, these error measures might not be the ideal choice here. Further to the potential problems discussed above, their sensitivity to outliers is another limitation [33,34]. As the evaluation of the models did not include testing for significant outliers, they might, if present, cause slight differences in forecast performance between the LASSO and RNN. Therefore, further model refinement should include testing for outliers in the predictions and, if necessary, error measures that are more resilient to outliers, such as MAPE [33].

Although the ordering strategy given by Equation 1 does consider current stock, it neglects the remaining shelf life of units in stock. Adapting orders to the expiry profile of current stock might be beneficial and should be investigated in further studies.

In future applications, the prediction and simulation environment presented here could be extended to other perishable goods whose consumption data show similar characteristics. The following data characteristics may be helpful in generalizing this approach to other problems: (1) the data of platelet demand investigated here are stationary in the presence of a trend, and (2) the data have a strong pattern of autocorrelation with weekly seasonality. From a practical point of view, the short shelf life and high variance of daily demand for platelets are important characteristics that should be considered to identify suitable problems for this approach. Our system could also be used to investigate possible optimization of the blood bank inventory, such as collection of platelets during weekends, by comparing savings in waste and shortage with additional staff costs.

### Conclusions

Both a LASSO model and an RNN with an LSTM layer can predict platelet demand at the UKA with high accuracy. This is in accordance with previous studies and further supports the generalizability of these models to different sites. The retrospective simulations of the blood inventory at the UKA presented here show that the predictions of both models enable a significant reduction in waste and shortage rates of platelets. Further research is needed to exploit the full potential of deep learning models for the prediction of platelet demand. Furthermore, there is a need for models that take into account ABO blood types in their predictions.

## Appendix

Multimedia Appendix 1 Quantitative data used to construct the figures and tables.

## Abbreviations

ANN: artificial neural network
ANOVA: analysis of variance
ARIMA: autoregressive moving averages
EHR: electronic health record
LASSO: least absolute shrinkage and selection operator
LSTM: long short-term memory
MAPE: mean absolute percent error
MSE: mean squared error
RMSE: root mean squared error
RNN: recurrent neural network
UKA: RWTH Aachen University Hospital
